# AlphaFold2's training set powers its predictions of some fold‐switched conformations

**DOI:** 10.1002/pro.70105

**Published:** 2025-03-25

**Authors:** Joseph W. Schafer, Lauren L. Porter

**Affiliations:** ^1^ National Library of Medicine, National Center for Biotechnology Information, National Institutes of Health Bethesda Maryland USA; ^2^ National Heart, Lung, and Blood Institute, Biochemistry and Biophysics Center, National Institutes of Health Bethesda Maryland USA

**Keywords:** alternative conformations, fold‐switching proteins, metamorphic proteins, protein structure prediction, structural bioinformatics

## Abstract

AlphaFold2 (AF2), a deep‐learning‐based model that predicts protein structures from their amino acid sequences, has recently been used to predict multiple protein conformations. In some cases, AF2 has successfully predicted both dominant and alternative conformations of fold‐switching proteins, which remodel their secondary and/or tertiary structures in response to cellular stimuli. Whether AF2 has learned enough protein folding principles to reliably predict alternative conformations outside of its training set is unclear. Previous work suggests that AF2 predicted these alternative conformations by memorizing them during training. Here, we use CFold—an implementation of the AF2 network trained on a more limited subset of experimentally determined protein structures—to directly test how well the AF2 architecture predicts alternative conformations of fold switchers outside of its training set. We tested CFold on eight fold switchers from six protein families. These proteins—whose secondary structures switch between α‐helix and β‐sheet and/or whose hydrogen bonding networks are reconfigured dramatically—had not been tested previously, and only one of their alternative conformations was in CFold's training set. Successful CFold predictions would indicate that the AF2 architecture can predict disparate alternative conformations of fold‐switched conformations outside of its training set, while unsuccessful predictions would suggest that AF2 predictions of these alternative conformations likely arise from association with structures learned during training. Despite sampling 1300–4300 structures/protein with various sequence sampling techniques, CFold predicted only one alternative structure outside of its training set accurately and with high confidence while also generating experimentally inconsistent structures with higher confidence. Though these results indicate that AF2's current success in predicting alternative conformations of fold switchers stems largely from its training data, results from a sequence pruning technique suggest developments that could lead to a more reliable generative model in the future.

## 1 INTRODUCTION

AlphaFold2 (AF2) is a deep‐learning‐based algorithm that predicts a protein's three‐dimensional structure from its amino acid sequence, often with high accuracy (Jumper et al., 2021). AF2's success at predicting one dominant conformation of an amino acid sequence has led to the development of algorithms for sampling conformational ensembles and alternative conformations with varying success (Alamo et al., 2022; Jing et al., 2024; Kalakoti & Wallner, 2025; Monteiro da Silva et al., 2024; Vani et al., 2023; Wayment‐Steele et al., 2024; Zheng et al., 2024). Some of these approaches focus on predicting both conformations of fold‐switching proteins, a class of globular proteins that remodel their secondary and/or tertiary structure in response to cellular stimuli (Porter & Looger, 2018). Fold switchers regulate biological processes and are associated with several human diseases, making them predictive targets of interest (Kim & Porter, 2021). Recent results indicate, however, that AlphaFold2 and AlphaFold3‐based approaches are weak predictors of fold switching (Abramson et al., 2024; Chakravarty & Porter, 2022; Chakravarty et al., 2024). Specifically, AlphaFold (AF)‐based methods tend to successfully predict one conformation accurately, consistently, and confidently but often fail to generate the other. We call the conformations that are predicted consistently and successfully “dominant” and the ones that AF tends to miss “alternative.” Though dominant conformations often correspond to those with the largest amount of coevolutionary information from the multiple sequence alignments AF uses to infer structure (Schafer & Porter, 2023), there are some exceptions (Chakravarty et al., 2025).

How AF2‐based methods predict alternative protein conformations remains unclear. The abstruse nature of neural network‐based predictions is a universal problem that has motivated approaches that render human‐interpretable explanations for how deep learning approaches work (Lundberg & Lee, 2017; Mehdi & Tiwary, 2024; Ribeiro et al., 2016). Two extreme explanations for how AF2 predicts alternative conformations have been proposed: the Generative Explanation, which posits that AF2 has learned enough folding principles to consistently and accurately predict alternative conformations outside of its training set, and the Associative Explanation, which posits that AF2's successful predictions of alternative conformations often depend on the structures it learned during training. In further detail, the Generative Explanation suggests that AF2 uses evolutionary couplings from its input multiple sequence alignment (MSA) to predict alternative conformations (Sala et al., 2023). In this framework, the information needed to specify a given fold is provided by the MSA, enabling AF2 to generate predictions of alternative protein conformations with high confidence regardless of whether they are in its training set. Alternatively, the Associative Explanation suggests that AF2 predicts alternative conformations from “memory” of structures learned during training (Bryant & Noé, 2024), allowing it to associate related input sequences and/or MSAs with these structures (Chakravarty et al., 2024). These inputs do not necessarily provide enough information to enable AF2 to predict a new fold accurately, however (Schafer et al., 2025). Instead, structures learned during training foster accurate predictions from sparse sequence information, limiting AF2's ability to reliably predict alternative conformations outside its training set (Chakravarty et al., 2024).

**FIGURE 1:**
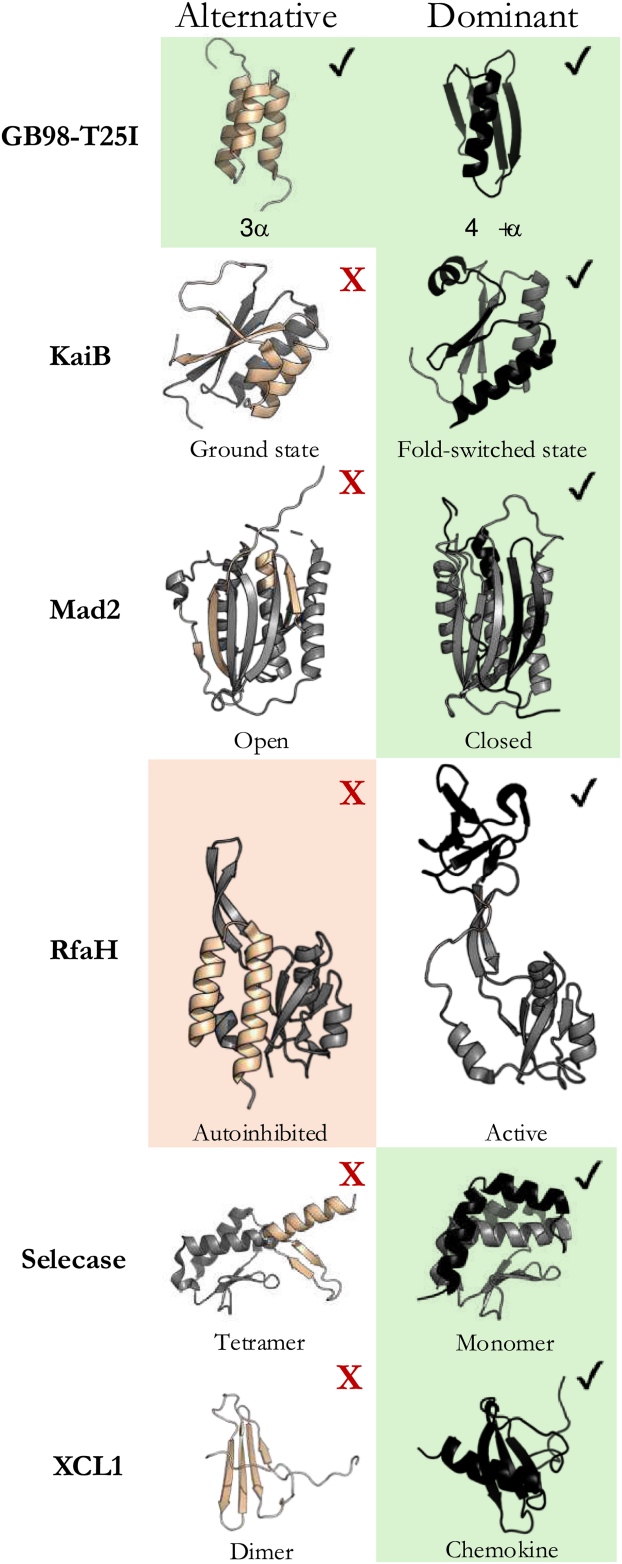
Alternative and dominant conformations of fold‐switching proteins from six families. CFold predictions from full MSAs denoted with check marks, those not predicted with red Xs; AlphaFold2 conformations predicted from full MSAs denoted with green and orange backgrounds. Green/orange means AF2 and CFold predicted the same/different conformations. Altnernative/dominant fold‐switching regions of proteins are beige/black.

While previous work has shown that AF2‐based predictions of both dominant conformations and alternative conformations similar to dominant can be generative (Bryant & Noé, 2024), here we test how well the AF2 network predicts both disparate conformations of fold switchers without exposure to the alternatives during training. Thus, we ran the network with a new set of model weights trained on the dominant conformations of a subset of fold switchers but not their alternative conformations (Figure 1; Table S1). Bryant and Noé trained this implementation of AF2 to predict alternative conformations and called it CFold (Bryant & Noé, 2024). CFold was benchmarked largely on rigid body motions and local conformational changes (Bryant & Noé, 2024). Here, we test it on eight previously untested fold switchers that undergo dramatic secondary structure remodeling and whose alternative conformations were not in the training set. Recent evidence suggests that AlphaFold has memorized the alternative conformations of these fold switchers during training, favoring the Associative Explanation in these cases. Here, we leverage the advance offered by CFold to test this possibility more directly. If the Generative Explanation holds for these fold switchers, we would expect CFold to predict their alternative conformations correctly and uniquely with high confidence, as AF2 does. If the Associative Explanation holds, we would expect CFold to fail to predict accurate alternative conformations with high confidence and/or to predict incorrect structures with high confidence, indicated by per‐residue local distance difference test (plDDT) scores ≥70 (or 0.7 for CFold). Any correct prediction with a confidence at or above this threshold was considered a success even if it was not the highest confidence prediction generated. In all cases tested, we find that CFold fails to predict alternative conformations of fold‐switching proteins outside of its training set reliably, supporting the Associative Explanation for alternative conformations of these fold switchers. However, predictions from a previously suggested sequence filtering technique (Schafer & Porter, 2023) suggest developments that could lead to a more reliable generative model in the future.

## 2 RESULTS

### 2.1 The dataset

CFold was tested on eight fold‐switching proteins, six of which are from different fold families (Figure 1; Table S1). The AF2 model on which CFold is built predicts both the dominant and alternative conformations of seven of these proteins (Chakravarty et al., 2024). However, previous work suggested that AF2 memorized the alternative conformations of six of these during training and the dominant conformation of Selecase. We added the eighth (GB98‐T25I) because AF2 predicts both of its experimentally observed conformations accurately and with high confidence. Because CFold was not trained on the alternative conformations of any of these proteins except GB98‐T25I, it allowed us to test the “memorization hypothesis” directly. Each protein family tested is described below.

- GB98‐T25I is an engineered fold‐switching protein that reversibly transitions between a 3‐α‐helix bundle and 4β + α fold (He et al., 2012).
- Three experimentally characterized KaiB proteins, whose fold switching helps regulate cyanobacterial circadian rhythms (Chang et al., 2015), were also evaluated (*S. elongatus* KaiB, *R. sphaeroides* KaiB, *T. elongatus* KaiB). KaiBs belong to a protein superfamily (large clade of diverse homologous sequences) composed of both single folders and fold switchers. All its members are expected to fold into a dominant thioredoxin‐like fold, but those that switch folds also assume an alternative ground state.
- RfaH is a bacterial transcription activator whose C‐terminal domain reversibly switches between a dominant β‐sheet fostering efficient translation and an alternative α‐hairpin that limits its activity to a specific DNA‐binding site (Burmann et al., 2012).
- The eukaryotic mitotic spindle protein Mad2 populates two conformations, open and closed (Mapelli et al., 2007). It forms a dimer consisting of one of each conformer that binds to other proteins involved in spindle assembly.
- Selecase—a concentration‐dependent fold switcher—assumes different conformations in different oligomeric states (Lopez‐Pelegrin et al., 2014)
- XCL1, the fold‐switching chemokine, assumes a monomeric fold involved in signaling and a dimeric fold that may recognize pathogens (Dishman et al., 2021).

It is important to note that “dominant” denotes the conformations that CFold predicts with the highest confidence from deep MSAs, not the conformation that the apo protein assumes most frequently under native conditions. Previous work shows that these “dominant” conformations are predicted from rich coevolutionary information (Schafer & Porter, 2023), explaining why CFold predicts them from full MSAs. A Foldseek search (van Kempen et al., 2024) indicated that CFold's training set contained all dominant conformations but only one alternative conformation of these fold‐switching proteins (GB98‐T25I; Table S2).

### 2.2 Full MSA sampling and random MSA subsampling produce training‐set conformations only

Full‐MSA sampling produced predictions of all structures in CFold's training set: all dominant and the one alternative (Figure 1). Neither full‐MSA sampling nor the random MSA subsampling suggested to be used with CFold (Bryant & Noé, 2024) produced any of the alternative conformations outside of CFold's training set (Figure S1), despite sampling 800 structures/protein (Table S3). Notably, while AF2 predicts RfaH's alternative helical conformation with high confidence from full MSA sampling, CFold consistently predicts its dominant β‐sheet conformation with high confidence and fails to predict its helical conformation with either full‐MSA sampling or suggested MSA subsampling (Figure S1). RfaH's helical conformation is not in CFold's training set, but it was likely in AF2's (Chakravarty et al., 2024). These results support previous observations suggesting that AF2 memorized RfaH's alternative helical conformation during training (Chakravarty et al., 2024).

### 2.3 Sequence clustering did not produce any successful predictions

Two other MSA‐based approaches were attempted to predict alternative conformations outside of CFold's training set: sequence clustering based on similarity (Wayment‐Steele et al., 2024) and subfamily filtering or pruning of the protein family tree (Schafer & Porter, 2023). These approaches—used to generate an additional 1100–3500 structures—differ because: (1) sequence clusters are shallower, containing ≤11 sequences while filtered subfamilies contain dozens to hundreds of sequences and (2) sequence clusters do not necessarily preserve phylogenetic order while subfamily‐filtered sequences do. Though AF2 successfully predicted the alternative conformations of some fold switchers using sequence clusters as input (Wayment‐Steele et al., 2024), CFold did not predict any conformations accurately and with high confidence from them (Figure S2). This result supports previous work indicating that the coevolutionary information from sequence clusters is insufficient to generate alternative conformations outside of AF2's training set (Schafer et al., 2025).

### 2.4 Subfamily filtering produced one successful prediction outside the training set and false positives

**FIGURE 2:**
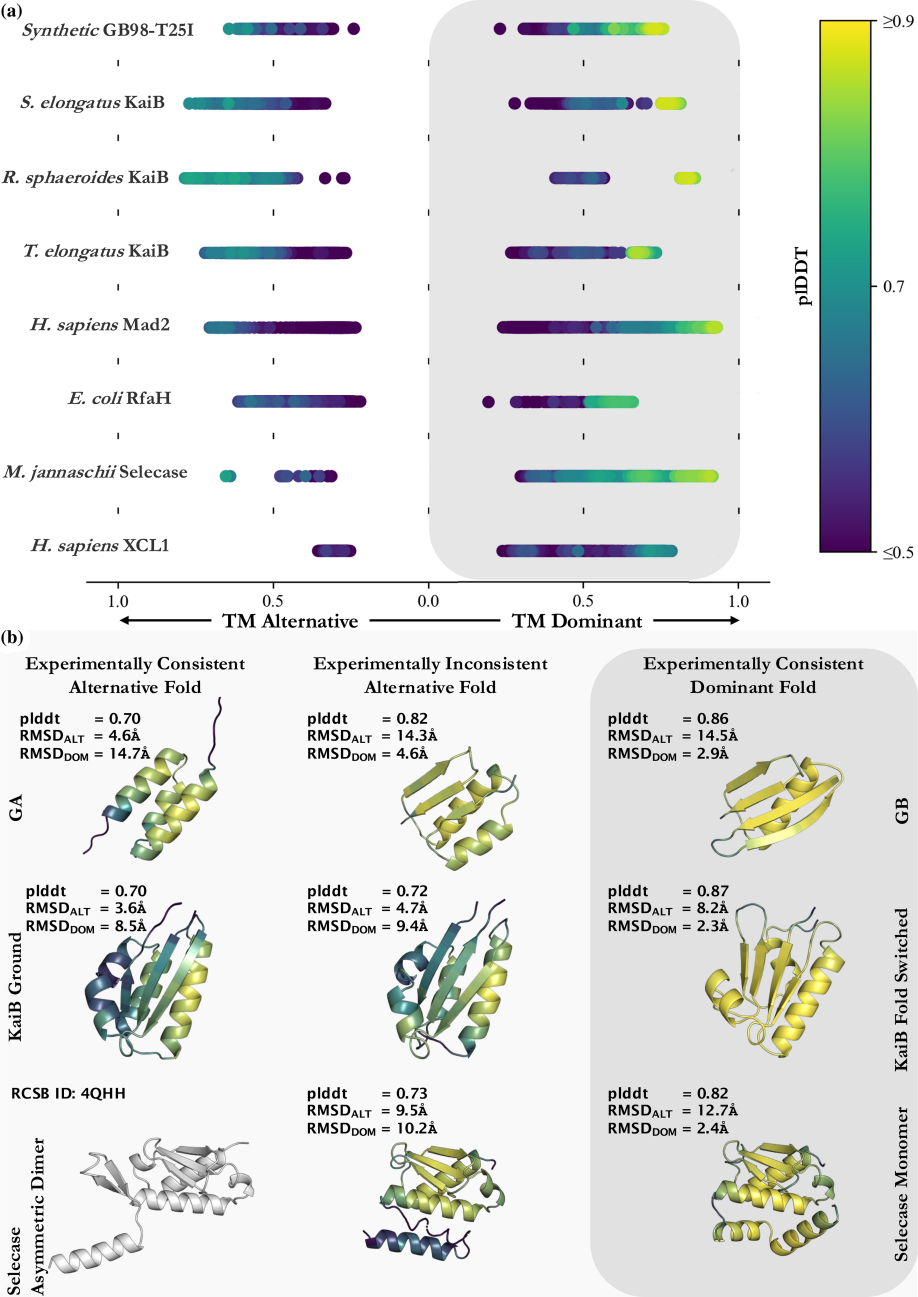
Pruning by sequence identity enables CFold to predict some experimentally consistent alternative structures with high confidence but also produces high‐confidence structures inconsistent with experiment. (a) TM‐scores for ensembles of predicted structures created with CFold for fold‐switching proteins. The predicted structure with the highest plDDT score defines the dominant predicted conformation for the CFold ensemble. Predicted structures within an ensemble are sorted by TM‐score: If the TM‐score is greater for the dominant conformation the value appears on the right side of panel a (gray box); otherwise, the value appears on the left‐side of panel a. All TM‐scores are colored by the predicted structures average plDDT score. (*RfaH calculations are sorted by TM‐score calculated for residues 118–155 to ensure conformations for this two‐domain protein are organized by the fold‐switching region. The TM‐score plotted is the TM‐score for the entire sequence compared to crystal structures. XCL1 calculations are on residues 1–65, the last 29 residues are unstructured and are not considered for TM‐score or plDDT score.) Experimentally determined structures used for comparison in order from top to bottom (alternative/dominant) are 2LHC/2LHD, 2QKE_A/1T4Y, 4KSO_A/8FWJ_M, 2QKE_A/1T4Y, 3GMH_L/2VFX_L, 5OND_A/6C6S_D, 4QHH_A/4QHF_A, 2HDM_A/4CVW_A. (b) High‐confidence predictions from Cfold generated ensembles with each structure's average plDDT score (top), RMSD to the alternative crystal structure (middle), and RMSD to the dominant crystal structure (bottom). The color scheme of these structures matches the colorbar shown in panel a. Each row shows predicted alternative and dominant structures consistent with experiment and one high‐confidence structure that is inconsistent. The experimentally determined gray alternative structure of Selecase was not predicted.

**FIGURE 3:**
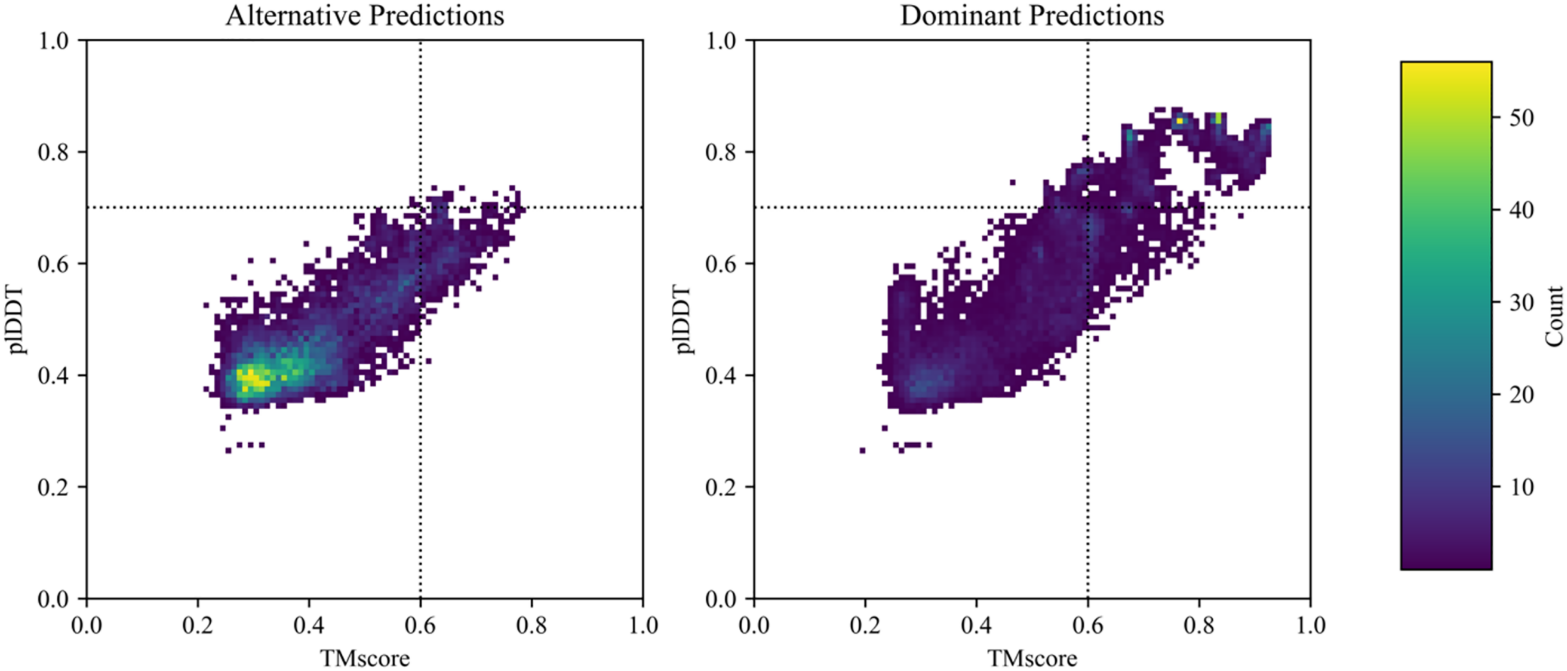
Two‐dimensional histograms of all predicted structures from *Synthetic* GB98‐T25I, *S. elongatus* KaiB, *R. sphaeroides* KaiB, *T. elongatus* KaiB, *H. sapiens* Mad2, *E. coli* RfaH, and *M. Jannashii* Selecase ensembles show distributions of plddt scores compared with TM scores. Predicted alternate structures appear on the left and dominant structures appear on the right (dominant/alternate defined in Figure 1). The color bar corresponds to the number of predicted structures within a bin. Successful predictions have TM scores ≥ 0.6 and plDDT scores ≥ 0.7; dashed lines are included as a visual aid. By these criteria, 27.9%/0.6% of dominant/alternative predictions are successful.

Subfamily filtering enabled more predictions of conformations loosely resembling some alternative conformations, but it was sufficient to capture the alternative conformation of only one KaiB variant accurately and with high confidence (Figure 2). This indicates that evolution has likely selected for many of these alternative conformations (Schafer & Porter, 2023), but the information provided by subfamily filtering is not sufficient to enable CFold to reliably predict alternative conformations of these fold switchers. For instance, CFold produced RfaH structures with alternative helical C‐terminal domains (CTDs), though these predictions were low‐confidence (plDDT < 0.6), and the structural quality was poor, with a best overall RMSD ≥ 12.0 Å (Figure S3a). Furthermore, incorrect structures with mixed α‐helix and β‐sheet CTDs were produced with higher confidence than the correct α‐helical form (Figure S3a). Thus, while subfamily filtering provides CFold with some information about RfaH's alternative fold, it is not sufficient to produce an accurate structure, nor does it enable recognition of the alternative structure with high confidence. Further, CFold failed to accurately predict the alternative conformations of Mad2, Selecase, and XCL1 with high confidence (Figure S3b), while AF2 has successfully achieved this for the alternative conformations of Mad2 and XCL1 (Chakravarty et al., 2024). It should be noted that our definition of a successful prediction is TM‐score ≥ 0.6; if Bryant and Noé's threshold of 0.8 is used, then zero alternative structures were predicted successfully (Figure 3).

While subfamily filtering enabled some successful predictions of alternative conformations, it also led to experimentally inconsistent predictions with high confidence. For instance, both experimentally consistent predictions of GB98‐T25I in Figure 2b flank an experimentally inconsistent structure predicted with a confidence (plDDT = 0.82) substantially higher than the experimentally consistent helical structure (plDDT = 0.70). This type of misprediction is particularly difficult to recognize because the two‐dimensional contact maps of the two predictions are nearly superimposable (Figure S4a). Thus, it is not obvious how coevolution could be used to discriminate between these two predictions, a situation that also occurs when using AlphaFold3 (Chakravarty et al., 2024). Similarly, CFold produces an experimentally inconsistent structure of KaiB with higher confidence than the experimentally consistent alternative structure (plDDT values of 0.7 and 0.72, respectively). Again, the two‐dimensional representations of these conformations were superimposable, rendering coevolution unable to differentiate between the two predictions (Figure S4b). AF2 does not make either of these two mispredictions with high confidence.

### 2.5 Mixed performance on single‐folding homologs of fold switchers

To determine whether CFold could distinguish between single folders and fold switchers, single‐folding homologs of GB98‐T25I, KaiB, and RfaH were also studied (Figures S5 and S6). CFold incorrectly predicted that GA98, GB98, and GB98‐T25I, L20A assume both the 3‐α‐helix bundle and a 4β + α fold. However, these sequences are not known to switch folds, though their sequences are highly identical to GB98‐T25I, which does. These findings show that AF2's architecture can incorrectly misclassify single folders as fold switchers with high confidence when their sequences are highly similar. CFold did not predict structures loosely resembling alternative folds for single‐folding KaiBs and NusG as single folders; however, indicating that AF2's architecture can sometimes distinguish between fold switchers and single folders with more disparate sequences.

## 3 DISCUSSION

Unlike AF2, which has been found to correctly predict fold switching with high confidence for all targets tested here except Selecase (Chakravarty et al., 2024), CFold predicts fold switching with high confidence for only GB98‐T25I and one KaiB variant while also producing false positives. Differences in the training set best explain these predictive discrepancies since AF2 and CFold have the same network architecture, and their makers used very similar training approaches (Bryant & Noé, 2024). The most direct explanation for AF2's unique, high‐accuracy predictions of these alternative conformations is that it recalls their structures from training (Bryant & Noé, 2024; Chakravarty et al., 2024). An alternative explanation is that AF2's more diverse training set helped it learn broader predictive principles than CFold's smaller set allows. Although this explanation is possible, previous research shows that the AF2 architecture performs nearly as well with a training set of 10,000 structures as it does with its full set of over 100,000 (Ahdritz et al., 2024). Given that CFold was trained on >50,000 structures, it is unclear if a larger dataset would yield additional insights. The most solid test would be to retrain AF2 on all conformations except for the alternative conformations presented here, but that requires very substantial computational resources.

Though many fold‐switching events are triggered by binding other proteins or molecules (Porter & Looger, 2018), those in our dataset are either monomeric or form homo‐oligomers (Table S1). The two exceptions are: (1) the β‐sheet fold of RfaH, which forms only upon binding RNA polymerase and a specific DNA sequence called *ops* (Zuber et al., 2019), and (2) the fold‐switched state of KaiB, which forms a complex with KaiA and KaiC (Tseng et al., 2017). Interestingly, CFold predicts the complexed forms of both RfaH and KaiB with high confidence but fails to predict RfaH's apo monomeric helical form and two of KaiB's ground‐state homotetrameric forms. This demonstrates that CFold predictions result from something other than biological context and is consistent with previous observations that adding protein binding partners and/or cofactors does not improve AF2 and AF3 predictions of fold switchers (Chakravarty et al., 2024). CFold also misses the alternative conformation of Mad2, which can be monomeric, and predicts unphysical forms of monomeric GB98‐T25I and KaiB‐Rs. These results further support our explanation that CFold's inability to predict alternative conformations of these fold switchers results from: (1) their lack of training‐set representation and (2) insufficient coevolutionary information. Since a protein's sequence is sufficient to provide information about both its fold(s) (Schafer & Porter, 2023; Toth‐Petroczy et al., 2016) and its homo‐oligomeric contacts (Ovchinnikov et al., 2014), the dataset used here is a fair test of CFold.

While CFold's limited ability to successfully predict alternative structures outside of its training set supports the Associative Explanation, it also suggests several future directions that may enable better predictions of yet‐to‐be‐discovered alternative conformations. First, since plDDT does not effectively discriminate between experimentally consistent and inconsistent structures predicted by either AF2 or CFold (Bryant & Noé, 2024; Chakravarty et al., 2024), alternative measures need to be developed. Physically based simulations and networks for reweighing members of predicted ensembles are promising avenues (Vani et al., 2024). Second, since subfamily filtering was the only approach that enabled predictions of alternative conformations outside the training set, methods to enhance that information may enable more accurate generative predictions of fold switching (Schafer & Porter, 2023). Finally, the problem of degenerate structural solutions to contact maps must be overcome. Since coevolution cannot always be used to discriminate between experimentally consistent and inconsistent predictions, incorporating physically based priors into the predictive network may aid that effort (Ishizone et al., 2024). Data and code supporting this work can be found at: https://github.com/porterll/CFold_AF2.

## AUTHOR CONTRIBUTIONS

**Joseph W. Schafer:** Investigation; writing – original draft; visualization; methodology; software; data curation; formal analysis. **Lauren L. Porter:** Conceptualization; methodology; visualization; writing – review and editing; funding acquisition; supervision.

## Supporting information


**Data S1:** Supporting Information.

## Data Availability

The data that support the findings of this study are openly available in CFold_AF2 at https://github.com/porterll/CFold_AF2.
